# Protamine dose to neutralize heparin at the completion of cardiopulmonary bypass can be reduced significantly without affecting post-operative bleeding

**DOI:** 10.1051/ject/2023026

**Published:** 2023-09-08

**Authors:** Min-Ho Lee, Matthew Beck, Kenneth Shann

**Affiliations:** 1 Perfusion Team, Massachusetts General Hospital 55 Fruit Street Boston MA 02114 USA; 2 Perfusion Services, Children’s Hospital of Philadelphia 3401 Civic Center Blvd. Philadelphia PA 19104 USA

**Keywords:** Protamine-to-heparin ratio, Protamine dose, Post-operative bleeding, Heparin protamine titration, Cardiopulmonary bypass

## Abstract

*Background*: Systemic anticoagulation with heparin during cardiopulmonary bypass (CPB) should be neutralized by protamine administration to restore normal hemostasis. Our previous study showed the protamine-to-heparin ratio (P-to-H) of 1:1 (1 mg protamine:100 IU circulating heparin; 1.0 Ratio) is likely an overestimation. Thus, we reduced the P-to-H in the HMS Plus Hemostasis Management System to 0.9:1 (0.9 Ratio) for 5 months and then to 0.8:1 (0.8 Ratio). We monitored post-operative (post-op) bleeding in the setting of reduced protamine dose (PD). *Methods*: We performed a retrospective study of 632 patients (209 for the 1.0 Ratio, 211 for 0.9 Ratio, 212 for 0.8 Ratio group) who underwent cardiac surgery to measure the reduction of PD and how it affects 24-hour (24 h) post-op chest tube output. We also analyzed the entire data set to explore whether further reduction of P-to-H is warranted. *Results*: While there was no difference in the indexed heparin dose among the three groups, we achieved a significant reduction in the indexed actual protamine dose (APDi) by 24% (0.9 Ratio) and 31% (0.8 Ratio) reductions compared to the 1.0 Ratio group. On average, APDi was 88 ± 22, 67 ± 18, and 61 ± 15 mg/m^2^ in the 1.0, 0.9, and 0.8 Ratio groups, respectively. We found no significant difference in 24 h post-op bleeding among the three groups. *Conclusion*: 1.0 Ratio at the completion of CPB is likely an excessive administration of protamine. With the stepwise reduction of PD, we observed no increase in post-op bleeding, which may indicate that no meaningful increase in heparin rebound occurred. In addition, further analysis of the entire data set demonstrates that a 0.75 Ratio is likely sufficient to neutralize the heparin completely.

## Introduction

At the completion of cardiopulmonary bypass (CPB), an optimal amount of protamine should be administered to restore normal hemostasis from systemic anticoagulation with heparin. With the administration of an optimal protamine dose (PD), neither residual heparin nor excessive protamine would be present. It is believed that protamine underdosing may cause heparin rebound to increase post-operative (post-op) bleeding [1–4] while excessive protamine dose has been linked to increased post-op bleeding as well [5–8] with increased activated clotting time (ACT) and decreased platelet function [9–13]. In addition, protamine has been shown to have anticoagulant properties in the absence of heparin and other side effects including anaphylactic response with hypotension, bradycardia, and pulmonary hypertension [7, 14–19].

There is no consensus on how to determine the optimal PD. One of the most common strategies is a fixed protamine-to-heparin ratio (P-to-H) with either the first heparin bolus (FHB) or total heparin dose (THD) during CPB, which does not account for heparin metabolism during CPB. Other strategies are developed to consider heparin metabolism and/or heparin concentration (HC) such as mathematical calculations of PD either using statistical or pharmacokinetic modeling of heparin metabolism over time or as a function of baseline and post-heparin ACTs; PD based on a measured HC, which should improve the accuracy of heparin neutralization and reduce PD [4, 19–25].

In general, it is considered that 1 mg protamine is sufficient to neutralize 100 international units (IU) of heparin (1.0 Ratio) [4, 26]. However, our previous study showed that the safe minimum PD to neutralize circulating heparin after CPB can be significantly lower than a 1.0 Ratio [27]. Based on this study, we changed the P-to-H in our HMS Plus Hemostasis Management System (HMS; Medtronic, Minneapolis, MN) to 0.9 Ratio for five months, then further reduced it to 0.8 Ratio without any complications. After this stepwise reduction of P-to-H, we performed a retrospective study to measure the actual protamine dose (APD) and how it affects 24-hour (24 h) post-op bleeding. In addition, we analyzed the entire data set to find out whether further reduction of P-to-H is warranted or not.

## Materials and methods

### Patient population

We reviewed the anesthesia and perfusion records of 313 patients from 1/1/2018 to 4/5/2018 who had cardiac surgery with CPB at Massachusetts General Hospital (MGH) with P-to-H of HMS being set at 1:1 (1.0 Ratio), 296 patients from 3/11/2020 to 7/15/2020 at 0.9:1 (0.9 Ratio), and 302 patients from 8/24/2020 to 11/25/2020 at 0.8:1 (0.8 Ratio). Patients with the following criteria were excluded for accurate analysis: missing or incomplete data and patients with a heart transplant, lung transplant, LVAD insertion, red blood cell transfusion between heparin dose response test (HDR) and FHB or deceased. We excluded 104, 85, and 95 patients, resulting in 209, 211, and 212 patients to be analyzed in the 1.0, 0.9, and 0.8 Ratio groups, respectively.

### Anticoagulation management

We performed an HDR test *in vitro* using the HMS to determine baseline ACT (bACT), and individual slope and calculated heparin concentration (CHC) to reach target ACT (tACT) before or right after induction of anesthesia. Our HMS is set to use IU of heparin and we used porcine heparin sodium (1000 USP units/mL) from SAGENT Pharmaceuticals (Schaumburg, IL) or FRESENIUS KABI (Lake Zurich, IL). Our tACT on CPB is ≥400 s and target HC (tHC) is ≥2.0 IU/mL. Our practice at MGH to determine FHB to go on CPB is described in detail elsewhere [27–29]. Additional doses of heparin were administered as necessary to maintain tACT and tHC during CPB.

To determine the PD required to neutralize heparin at the completion of CPB, we measure the HC 5–10 min after removal of the cross-clamp. P-to-H is expressed as either mg protamine:100 IU heparin or a Ratio such as 1:1 or 1.0 Ratio throughout this manuscript. P-to-H in our HMS was set at 1:1 (1 mg protamine per 100 IU heparin), 0.9:1, or 0.8:1 as indicated for the calculation of PD. HMS calculates and provides three protamine doses: patient, pump, and total protamine doses. The calculated total protamine dose (CTP) is the calculated pump dose plus the calculated patient protamine dose (CPP).

CPP (mg) = measured HC (IU/mL) × Total Blood Volume (TBV, mL) × P-to-H (1 mg/100 IU, 0.9 mg/100 IU, or 0.8 mg/100 IU)

Pump protamine dose (mg) = measured HC (IU/mL) × Pump Volume (1300 mL) × P-to-H (1 mg/100 IU, 0.9 mg/100 IU, or 0.8 mg/100 IU)

CTP (mg) = CPP + pump protamine dose

Our guideline recommends using CPP based on our previous study [27]. However, it is at the discretion of the perfusionist, anesthesiologist, and surgeon to determine the actual protamine dose (APD) for each patient based on HC and other considerations including the time between the last heparin protamine titration (HPT) and protamine administration. We administer protamine sulfate (10 mg/mL, FRESENIUS KABI, Lake Zurich, IL) by bolus with a test dose (~10 mg) first to observe any adverse reaction, then up to a half dose, remove the arterial cannula and give the remainder of PD. Heparin neutralization by protamine is verified by measuring ACT and HC using a red HPT cartridge three minutes after the completion of protamine infusion. After we confirm heparin neutralization, we do not administer additional protamine prophylactically to prevent potential heparin rebound.

### Data analysis

We used Microsoft Office Excel 365 (Microsoft Corporation, Redmond, WA, USA) to perform data input, calculations, statistical analysis (Chi-square test of independence, *p*; Student’s t-test, *p*), and the box and whisker plots. We employed heparin and protamine doses indexed to a body surface area (BSA) such as indexed FHB (FHBi), indexed THD (THDi), and indexed APD (APDi) for objective comparisons among three different groups. We calculated BSA and TBV following Du Bois and Du Bois, 1916 [30] and Allen et al, 1956 [31], respectively.

## Results

### Reduction of protamine-to-heparin ratio significantly reduced the actual protamine dose

Our previous study strongly indicated that P-to-H at 1 mg protamine/100 IU heparin (1:1 or 1.0 Ratio) of the circulating heparin at the completion of CPB is likely an overestimation (Table 1) [27]. Based on the study, we changed the P-to-H in our HMS to 0.9 mg protamine/100 IU heparin (0.9:1 or 0.9 Ratio) for five months, then further reduced to 0.8 mg protamine/100 IU heparin (0.8:1 or 0.8 Ratio).

The average calculated patient protamine dose (CPP) reduced significantly from 152 ± 53 (1.0 Ratio) to 133 ± 46 (0.9 Ratio) and 116 ± 37 mg (0.8 Ratio), which is reflected in the actual protamine dose (APD) of 174 ± 53 (1.0 ratio) to 136 ± 46 (0.9 Ratio) and 121 ± 36 mg (0.8 Ratio; Table 1a) with strong statistical differences (*p*, 0.00; Table 1a). On average, the ratio of APD to FHB was 0.46:1 and that of APD to THD was 0.32:1 in the 0.8 Ratio group compared to 0.67:1 and 0.47:1 respectively in the 1.0 Ratio group (Table 1b).

**Table 1a:** Patient information, Protamine doses, ppACT/bACT distribution.

	1.0 Ratio^a^ (Avg ± SD)	*P*-value (1.0 vs 0.9)	0.9 Ratio^a^ (Avg ± SD)	*p*-value (0.9 vs 0.8)	0.8 Ratio^a^ (Avg ± SD)	*P-*value (1.0 vs 0.8)
Height (cm)	171.6 ± 10.2	0.15	173.1 ± 11.2	0.11	171.5 ± 9.9	0.90
Weight (kg)	84.6 ± 17.1	0.04	88.4 ± 21.1	0.01	83.7 ± 17.9	0.59
BMI	28.6 ± 4.9	0.18	29.4 ± 6.1	0.07	28.4 ± 5.2	0.57
BSA (m^2^)	1.97 ± 0.23	0.05	2.02 ± 0.27	0.02	1.96 ± 0.23	0.62
Platelets (counts/nL blood)	225 ± 65	0.59	228 ± 63	0.63	225 ± 68	0.97
CPB time (min)	135 ± 60	0.03	148 ± 61	0.23	141 ± 65	0.35
Cross-Clamp Time (min)	97 ± 48	0.04	106 ± 43	0.43	102 ± 47	0.22
First Heparin Bolus (FHB, IU)	25959 ± 7003	0.16	26938 ± 7249	0.06	25693 ± 6129	0.68
Total Heparin Dose (THD, IU)	38007 ± 10823	0.33	39033 ± 10691	0.18	38427 ± 9853	0.68
First Hep. Conc.^b^	3.5 ± 0.9	0.42	3.5 ± 0.7	0.94	3.5 ± 0.6	0.37
Last Hep. Conc.^c^	2.7 ± 0.7	0.01	2.5 ± 0.6	0.13	2.6 ± 0.6	0.25
Cal. Patient Protamine dose (CPP, mg)	152 ± 53	0.00	133 ± 46	0.00	116 ± 37	0.00
Cal. Pump Protamine dose (mg)	35 ± 9	0.00	29 ± 7	0.00	27 ± 6	0.00
Cal. Total Protamine dose (CTP, mg)	187 ± 60	0.00	163 ± 51	0.00	143 ± 41	0.00
Actual Protamine Dose (APD, mg)	174 ± 53	0.00	136 ± 46	0.00	121 ± 36	0.00
Baseline ACT (bACT, sec)	132 ± 16	0.94	131 ± 20	0.55	132 ± 14	0.54
Post-Protamine ACT (ppACT)	112 ± 15	0.00	119 ± 18	0.36	118 ± 14	0.00
ppACT/bACT (%)	86 ± 12	0.00	92 ± 18	0.07	90 ± 13	0.00

a 1.0 Ratio 209 patients, 0.9 Ratio 211 patients, 0.8 Ratio 212 patients.

b HC measured by HPT prior to CPB after FHB.

c HC measured by HPT 5-10 minutes after cross-clamp comes off.

**Table 1b:** Indexed heparin and protamine dose.

	1.0 Ratio^a^ (Avg ± SD)	*P*-value (1.0 vs 0.9)	0.9 Ratio^a^ (Avg ± SD)	*p*-value (0.9 vs 0.8)	0.8 Ratio^a^ (Avg ± SD)	*P-*value (1.0 vs 0.8)
BSA (m^2^)	1.97 ± 0.23	0.05	2.02 ± 0.27	0.02	1.96 ± 0.23	0.62
First Heparin Bolus (FHB, IU)	25959 ± 7003	0.16	26938 ± 7249	0.06	25693 ± 6129	0.68
APD/FHB (%)	0.67 ± 0.15	0.00	0.50 ± 0.13	0.00	0.46 ± 0.11	0.00
First Heparin Bolus/BSA(FHBi, IU/m^2^)	13130 ± 3919	0.59	13283 ± 2741	0.37	13056 ± 2410	0.78
Total Heparin Dose (THD, IU)	38007 ± 10823	0.33	39033 ± 10691	0.18	38427 ± 9853	0.68
APD/THD (%)	0.47 ± 0.10	0.00	0.36 ± 0.10	0.00	0.32 ± 0.7	0.00
Total Heparin Dose/BSA(THDi, IU/m^2^)	19220 ± 4693	0.44	19251 ± 4144	0.46	19549 ± 4056	0.94
First Hep. Conc.	3.5 ± 0.9	0.42	3.5 ± 0.7	0.94	3.5 ± 0.6	0.37
Actual Protamine Dose (APD, mg)	174 ± 53	0.00	136 ± 46	0.00	121 ± 36	0.00
APD, % Reduction from 1.0 Ratio			22%		30%	
Actual Protamine Dose/BSA (APDi, mg/m^2^)	88 ± 22	0.00	67 ± 18	0.00	61 ± 15	0.00
APDi, % Reduction from 1.0 Ratio			24%		31%	

a Protamine:Heparin ratio expressed as (Protamine dose in mg x 100)/Heparin in IU.

### Indexed Heparin and protamine doses are required for objective comparisons among independent groups

While other characteristics such as height, platelets, and the first HC were not statistically different, we found that the 0.9 Ratio group had a significant difference from the 0.8 and 1.0 Ratio groups in the weight and BSA (Table 1a). Thus, to have a fair comparison of the heparin and protamine doses among the three groups, we decided to compare heparin and protamine doses indexed to BSA.

As shown in Table 1b, FHBi and THDi became closer among the three groups with the increase in *p* values compared to FHB and THD, indicating less difference exists in FHBi and THDi than in FHB and THD. This data suggests that we should use the indexed values when we discuss the heparin and protamine doses because the sizes of patients can be significantly different in two independent populations.

While there is no difference in FHBi and THDi among the three groups, we achieved a significant reduction in APDi. There were 24% (0.9 Ratio) and 31% (0.8 Ratio) reductions in APDi compared to the 1.0 Ratio group (Table 1b). On average, we administered 61 mg/m^2^ protamine in the 0.8 Ratio group where all but 5 patients had no residual heparin based on an HPT after protamine administration. However, three of the 5 had lower post-protamine ACT (ppACT) than bACT, possibly indicating errors in the HPT test (data not shown). This data is essentially identical to the 1.0 Ratio group where four patients had residual heparin but two of the 4 had lower ppACT than bACT ([27], data not shown). In addition, the averages of ppACT are consistently lower than bACT in all three groups (Table 1a) as shown in our previous study [27]. These data suggest that CPP based on HPT at P-to-H of 0.8:1 is sufficient to neutralize heparin in almost all cases at the completion of CPB and likely has less excessive protamine after heparin neutralization.

### The significant reduction of protamine dose did not affect post-op bleeding

It is possible to have a more pronounced heparin rebound and increased post-op bleeding with a significant reduction in APD, which was approximately a 30% reduction from the 1.0 to 0.8 Ratio groups (Table 1b). Since we do not measure ACT, HC by HPT, or anti-factor Xa post-op, we do not have direct evidence of the occurrence and/or measurement of heparin rebound with the reduction of APD. Thus, we decided to compare the 24hr chest tube output and RBC transfusion requirement among the three groups. If the reduction of APD resulted in significantly higher heparin rebound clinically and increased anticoagulant activity, increased 24hr post-op bleeding and a possible increase in RBC transfusion requirement may occur.

**Figure 1:**
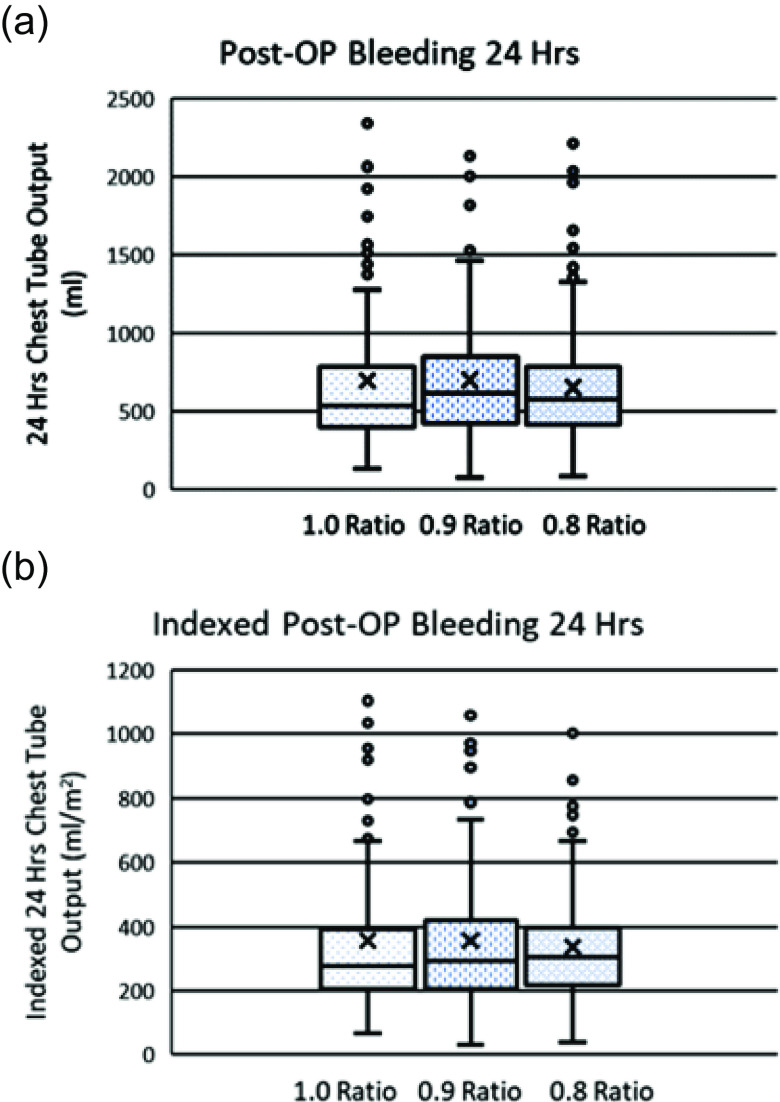
Non-Indexed and indexed 24-hour post-op bleeding. Non-indexed (a) and indexed (b) total chest tube outputs of 24hr post-op of the three groups are shown as box and whisker plots. *p* values of the chi-square test of independence of the non-indexed median (a) between any two groups are greater than 0.05 (Table 2). *P*-value of the chi-square test of independence of the non-indexed median (a) between 1.0 and 0.8 is 0.14 while that of the indexed (b) is 0.02 (Table 2). Outliers greater than 2500 ml (a) and 1200 ml (b) are not shown, respectively.

Chest tube output is used as a measurement of bleeding. As shown in Table 2, there is no significant difference in 24hr post-op bleeding (Chest tube (mL), mean ± standard deviation (SD)) among the three groups. A slight increase occurred in the 0.9 and a slight decrease in the 0.8 compared to the 1.0 Ratio group. However, these differences are not statistically significant as shown by high *p*-values (Table 2). Since the 0.9 Ratio group has a significantly higher BSA than the 1.0 and 0.8 Ratio groups, we also compared 24hr post-op bleeding indexed to BSA (Chest tube/BSA; Table 2). Means of the indexed 24hr post-op bleeding became closer among the three groups with slightly increased *p* values compared to non-indexed ones. The median, 1^st^ quartile, and 3^rd^ quartile of the non-indexed and indexed were all close to each other among the three groups (Table 2; Figure 1). Only the *p*-value of the chi-square test of independence of the indexed medians between the 1.0 and 0.8 Ratio groups shows that the indexed medians between the two groups are different statistically. However, that of the non-indexed medians and *p* values of non-indexed and indexed means between the two groups show that there is no significant difference (Table 2; Figure 1). Consistent with the 24 h post-op bleeding, we have not found a significant difference in the 24 h RBC transfusion requirement among the three groups (Table 2).

**Table 2:** 24-hour post-op bleeding and Red Blood Cell transfusion.

	1.0 Ratio	*p* (1.0 vs 0.9)	0.9 Ratio	*p* (0.9 vs 0.8)	0.8 Ratio	*p* (1.0 vs 0.8)
Chest Tube (ml), mean ± sd	692 ± 520	0.87*	700 ± 458	0.21*	650 ± 348	0.33*
Chest Tube (ml), q1	400		420		414	
Chest Tube (ml), median	535	0.06^	610	0.47^	570	0.14^
Chest Tube (ml), q3	780		848		776	
Chest Tube/BSA (ml/m^2^), mean ± sd	354 ± 264	0.99*	354 ± 259	0.36*	335 ± 180	0.38*
Chest Tube/BSA (ml/m^2^), q1	206		207		217	
Chest Tube/BSA (ml/m^2^), median	273	0.49^	292	0.52^	303	0.02^
Chest Tube/BSA (ml/m^2^), q3	388		415		394	
RBC transfusion (unit)	0.1 ± 0.6	0.09*	0.3 ± 1.0	0.44*	0.2 ± 0.6	0.21*

* *p*-value of Student’s t-test.

^ *p*-value of chi-square test of independence.

### Can protamine to heparin ratio be further reduced below 0.8:1?

Previously, we showed that ppACT/bACT (%) can be used as a reliable indicator to analyze a safe minimum protamine dose, 80–90% of ppACT/bACT is a good indicator of proper neutralization of heparin and 94% of CPP on average can be a safe minimum protamine dose to neutralize heparin at the completion of CPB [27].

We combined all three groups and categorized them into APD/CPP distribution to find the smallest possible P-to-H. All CPPs are recalculated in P-to-H of 1:1 for this analysis. As expected with three groups combined, the averages of APD/CPP are widely distributed as 75–141% of CPP (Table 3). However, ppACT/bACT are similar in all groups (85–92% on average) and no statistically significant difference exists in neighboring groups except between 81–90% and 91–100% (Table 3). The average P-to-H of the smallest APD/CPP group (44–80%) is 0.75:1 of circulating heparin.

**Table 3:** APD/CPP distribution of all data combined.

APD/CPP*	44–80%	*p*	81–90%	*p*	91–100%	*p*	101–110%	*p*	111–120%	*p*	121–130%	*p*	131–192%
# Pt (%)	122 (19%)		151 (24%)		119 (19%)		72 (11%)		65 (10%)		57 (9%)		46 (7%)
Avg	75 ± 2%	0.00	86 ± 3%	0.00	95 ± 3%	0.00	105 ± 3%	0.00	116 ± 3%	0.00	125 ± 3%	0.00	141 ± 14%
ppACT/bACT	92 ± 17%	0.91	91 ± 16%	0.04	88 ± 14%	0.24	90 ± 17%	0.09	86 ± 12%	0.65	85 ± 9%	0.47	85 ± 14%

* CPP is calculated in P-to-H of 1:1.

## Discussion

Adequate neutralization of heparin by protamine at the completion of CPB is important to minimize bleeding after CPB. Although protamine is an effective means of reversing heparin, adverse events, including hypotension, pulmonary edema, and anaphylaxis, can occur. In addition, free protamine with excessive doses has been linked to increased post-op bleeding. Administering an optimal dose of protamine to neutralize heparin completely without the excessive free protamine should help to minimize these adverse events while ensuring hemostasis [5–8, 14–19, 32–33].

Since there is no available measurement of free circulating protamine, protamine dosing has been largely empirical. It is usually expressed as protamine: heparin ratio. A common recommendation is a fixed 1:1 (1 mg protamine to every 100 IU of heparin) or 0.8 ratios based on FHB needed to establish therapeutic anticoagulation to go on CPB. Other strategies are based on THD during CPB or the latest circulating HC. Thus, it is important to clarify what the base of the ratio is to discuss the protamine to heparin ratio [4, 19–25].

Based on our previous study showing that the 1.0 Ratio of the circulating heparin at the completion of CPB is likely an overestimation [27], we changed the P-to-H in our HMS to a 0.9 Ratio first, then further reduced to 0.8 Ratio. This change resulted in a 31% reduction of APDi from the 1.0 to 0.8 Ratio groups (APDi from 88 to 61 mg/m^2^). This translates to, on average, our P-to-H was 0.32:1 for THD and 0.46:1 for FHB, which is significantly lower than the conventional fixed ratio of 1:1 or 0.8:1 of FHB (Table 1b).

With the significant reduction of APDi, it is possible to increase post-op bleeding due to heparin rebound. Since we do not perform an anti-factor Xa assay post-op, we compared the first 24 h post-op bleeding with measured chest tube output among the three groups. There were no significant changes in mean or median chest tube output. Quartiles 1 and 3 are also comparable, suggesting that the degree of outliers among the three groups is similar (Table 2, Figure 1). Consistent with this finding, there was no significant change in RBC transfusion during the first 24 h post-op among the three groups. All these data suggest that no clinically meaningful heparin rebound occurred with the significant reduction of APDi.

Our data is consistent with the previous findings. Heparin rebound might contribute to post-op bleeding and increased transfusion requirements. However, reported heparin rebound with anti-factor Xa assay has been minimal (less than 0.06 unit/mL) [1, 4] and heparin rebound might not be as important as a cause of post-op bleeding as previously thought. No correlation between post-protamine HC and post-op bleeding was found. Others showed that different P-to-H ratios are associated with a similar incidence of heparin rebound or no heparin rebound in the post-op period [1, 3, 4, 7, 34].

Our study shows that, in the discussion of heparin and protamine doses of two or more independent groups, it is important to use the doses indexed to BSA since a larger total blood volume is expected with the larger BSA. This is shown in our data that the 0.9 group has a larger BSA than the 1.0 and 0.8 groups, which is statistically significant. This resulted in a significant increase in FHB in the 0.9 compared to the 1.0 and 0.8 Ratio groups. However, there was essentially no change in the FHBi and the first HC 3 min after FHB (Table 1b), which strongly supports that the indexed heparin and protamine doses should be used when comparing two or more independent groups.

In addition, further analysis of our entire data set shows that there is more room to reduce P-to-H to 0.75 mg protamine to 100 IU circulating heparin to completely neutralize the circulating heparin (Table 3), which is consistent with our previous findings [27] and likely decreases the probability of excessive protamine and side effects of protamine.

Limitations: PD can be dependent on the tACT on CPB. Our tACT on CPB is 400 s and tHC is 2.0 IU/mL or higher [27–29]. It is likely that more heparin would be administered with a higher tACT on CPB, resulting in a higher circulating HC. Thus, a higher APD than the one found in this study may be needed with the higher tACT. We cannot completely rule out a remote possibility that significant differences exist in the pro-coagulant transfusions such as FFP, platelet, etc. among the three groups.

## Data Availability

All available data are incorporated into the article.
